# Supplementary material for: Relationship between Glucagon-like Peptide-1 Receptor Agonists and Cardiovascular Disease in Chronic Respiratory Disease and Diabetes
Source: Biomedicines. 2024 Feb 22;12(3):488. doi: 10.3390/biomedicines12030488 (PMC10968458; doi:10.3390/biomedicines12030488)
Supplement: Supplementary file 1 [file biomedicines-12-00488-s001.zip › biomedicines-2843625-supplementary.pdf]

Supplemental Figure S1. Flow chart of patients selection

Chronic respiratory diseases (CRD) with diabetes (DM) (CD cohort ,N=116947) During the study cohort (1998 01.01-2018-12.31) Inclusion cteteria : aged ≧20 years. If a patient had at least these two disease diagnosis requirements within 1 year of follow-up, a washout period of minimum 3 years was used to ensure that the patient was newly diagnosed

Excluded creteria (n= 7378): patients with whose sex were unknown, were not Taiwanese citizens, or younger than 20 years , had no CRD-related prescription requirements or related management or procedure 1 year after the first CRD diagnosis; had a disease history of CRD; were diagnosed with DM before the first CRD diagnosis or no antidiabetic prescription statement or DM–related management or procedure, and received GLP-1 RAs <3 time.

CD cohort with GLP-1 RAs use (n=31101) The index date indicates when GLP-1 RA was first prescribed.
The length of exposure to CD treatment with GLP-1 RA therapy was calculated as the time from the index date to the occurrence of the study outcomes or the final GLP-1 RA prescription During the observation period. A 90-day interval between prescription refills indicated therapy discontinuation. Patients were eliminated from the study when GLP-1 RA treatment was discontinued, a treatment switch occurred between the use of GLP-1 RA, or patients withdrew from the National Health Insurance program.

CD cohort without GLP-1 RAs use (n=78468)

Propensity score matching 1:1

N=15801

N=15801

odels

Adjusting for age, gender, comorbidities of hypertension, hyperlipidemia, chronic renal disease, gout, tobacco dependence-related, venous thrombosis. depression or substance related and medications of alpha glucosidase inhibitor (AGI), metformin, insulin, dipepidyl peptidase 4 (DPP-4) inhibitor, meglitinides, thiazolidinedione (TZD), sulphonylurea, statin, antidrepressants, antihypertensive, antithrombotic and psychotherapy

Supplemental Table S1. Full names of ICD-9-CM or ICD-10-CM diagnoses.

**Cohort definition**		
**Chronic respiratory disease**		
491, 492 and 496	COPD	J40-J44
493	Asthma	J45
494	Bronchiectasis	J47
135, 237.7, 272.2, 277.3, 277.8,500–505, 506.4, 508.1,	ILD	J80-J84

508.8, 515–516, 446.21, 446.4, 495, 517.2, 517.8, 518.3, 555, 710, 714.81, 720, and 759.5		
327.23<br>780.57	Sleep apnea	G47.3
416.0, 416.8,416.9	Pulmonary hypertension	I26-I28
180-182	Lung cancer	C34.91
010-012	Pulmonary tuberculosis	A15
250	DM	E09-E11, E13<br>A 181
 518.83<br>518.84	Respiratory failure<br>Chronic respiratory failure<br>Acute and chronic respiratory failure	J96<br>J96.10<br>J96.2
**Major outcome**		
**ICD-9CM**	**Full name**	
410-414	Ischaemic<br>Heart disease	I20-I25
410	Acute myocardial<br>infarction	I20-I22
411	Other acute and subacute forms of ischemic heart disease	I24
412	Old myocardial infarction	I25.2
413	Angina pectoris	I20
414	Other forms of chronic ischemic disease	I25
425	Cardiomyopathy	I42
426	Conduction disorder	I45.89
427	Cardiac arrhythmia	I49
428	Heart failure	I50.9
430	Subarachnoid haemorrhage	I60
431	Intracerebral haemorrhage	I61
433	Occlusion and stenosis of precerebral arteries	I63
434	Occlusion of cerebral arteries	I63
435	Transient cerebral ischemia	I63
436	Acute but ill-defined cerebrovascular disease	I63
**Comobordities**		
401-405	Hypertension	I10-I15, I16
272	Hyperlipidemia	E78
585	Chronic renal disease	N18
451.0 453.89	Venous thrombosis	I26, I82, 0082, 0223, 0871, 0882, I81
274	Gout	M10.9
305.1, 305.11, 305.12, and 305.13<br>649.01	Tobacco dependence -related	Z77.22 F17.Z72.0<br>P96.81<br>Z87.891<br>Z57.31
311, 296.3	Depression	F32.9
291, 292<br>303, 304, 305	Substance-related disorders	F10–F19, F55
**Drugs(Medicatins)**		

	**ATC**	**FULL NAMES**	

Bronchodilators	R03AC12<br>R03AC13	LABAs<br>Long-acting beta2 agonists<br>Salmeterol<br>Formoterol	
	R03BB04	LAMAs<br>long-acting muscarinic antagonists<br>Tiotropium bromide	
	R03AC02<br>R03AC03<br>R03AC04	SABAs<br>Short-acting beta2 agonists<br>Salbutamol<br>Terbutaline<br>Fenoterol	
	R03BB01	SAMAs<br>Short-acting muscarinic antagonists<br>Ipratropium bromide	
Steroids	R03BA01, R03BA02,<br>R03BA05,<br>R03BA08	ICSs<br>Inhaled corticosteroids<br>Beclometasone<br>Budesonide<br>Fluticasone<br>Ciclesonide	
	D07AC15,<br>D07AC17, H02AB02,<br>H02AB04, H02AB06,<br>H02AB08<br>R01AD05, S01BA02,	OSs<br>Oral steroids<br>Beclometasone<br>Fluticasone<br>Dexamethasone<br>Methylprednisolone<br>Prednisolone<br>Triamcinolone<br>Budesonide<br>Hydrocortison	
Antiarrhythmia	C01B	Anti-arrhythmic drugs	
Antidepressionants	N06AB03	Fluoxetine	
Benzodiazepines (BZDs)	N05BA12	Alprazolam	
	N05BA17	Fludiazepam	
	C10AA	Statins	
	C03	Diuretics	
	B01A<br>B01AA<br>B01AB<br>B01AC<br>B01AD<br>B01AE<br>B01AF	Anticoagulation<br>Vitamin K antagonists<br>Platelet aggregation inhibitors excluding heparin<br>Enzymes<br>Direct thrombin inhibitors<br>Direct factor Xa inhibitors	
	C07AB03<br>C07AB07<br>C07AB02	Beta blockers<br>(Cardioselective)<br>Atenol<br>Bisprol<br>Metoprolol	

	C09AA	Angiotensin converting enzyme inhibitors	
	C09CA	Angiotensin receptors blockers	
	C08	Calcium channel blockers	
	A10B	Antidiabetes	
	A10BA	Biguanides (Glucophage) ♥	
	A10BB<br>A10BB01<br>A10BB02<br>A10BB03<br>A10BB06<br>A10BB31<br>A10BB07<br>A10BB09<br>A10BB12<br>A10BB04	Sulfonylureas ¥<br>Glibenclamide<br>Chlorpropamide<br>Tolbutamide<br>Carbutamide<br>Acetohexamide<br>Glipizide<br>Gliclazide<br>Glimepiride<br>Glibornuride	
	A10BC<br>A10BD	Sulfonamides (heterocyclic)<br>Combinations of oral blood glucose lowering drugs	
	A10BF	Alpha glucosidase inhibitors (Acarbose)	
	A10BG	Thiazolidinediones<br>Rosiglitazone (Avandia)<br>Pioglitazone (Actos)♥	
	A10BH<br><br>A10BH05<br>A10BH03<br>A10BH02<br>A10BD25<br>A10BD07<br>A10BD08	Dipeptidyl peptidase 4 (DPP-4) inhibitors ♥<br>Linagliptin<br>Saxagliptin<br>Vildagliptin<br>Saxagliptin/Dapagliflozin<br>Sitagliptin/Metformin<br>Vildaglitin/Metformin	
	A10BJ<br><br>A10BJ01<br>A10BJ0<br>A10BJ05	Glucagon-like peptide-1 (GLP-1) analogues♥<br>Exenatide<br>Liraglutide<br>Dulaglutide	
	A10BK<br><br><br>A10Bk01<br>A10BK03<br>A10BK02<br>A10BD15<br>A10BD22	Sodium-glucose co-transporter 2 (SGLT2) inhibitors♥♥<br>*Dapagliflozin*<br>Empagliflozin<br>Canagliflozin<br>Dapagliflozin/Metformin<br>Dapagliflozin/Saxagliptin	
	A10BX	Other blood glucose lowering drugs, excl. insulins	
	A10BX02	Repaglinide (NovoNorm)	
	A10A	Insulins and analogues	
	A10AB	Insulins and analogues for injection, fast-acting	

	A10AC	Insulins and analogues for injection, intermediate-acting	
	A10AD	Insulins and analogues for injection, intermediate- or long-acting combined with fast-acting	
	A10AE	Insulins and analogues for injection, long-acting	
	A10AF		
**Immunosupprassants**			
	L01AA01	Cyclophosphamide (CYC)	
	L04AX01	Azathioprine(AZA)	
	L04AX03	Methotrexate(MTX)	
	L04AB06	TNF-α antagonists	
	L04AD01	Cyclosporine	
**Chemotheray**			
	L01XE02	Iressa	
	L01EB02	Tarceva	
	L01BC05	*Gemcitabine*	
	L01XA01	Cisplatin	
**New asthma drugs**			
	R03DX09	Mepolizumab	
	R03DX05	Omalizumab (Xolair)	
**Pulmonary fibrosis**			
	KF29M1		
	L01EX09	Nintedanib (Ethanesulfonate)	
**Vacination**			
	J07BB, 07BB01	Influenza	
	J07BB02, J07BB03	vaccination	
**Procedures**			
Chest-X-ray	32001C- 32002C		
CT-related	33070B ,33071B, 33072B, 33103B		
Pulmonary function-related teat	17001C, 17002B, 17003C, 17006C, 17004B-17018B, 17019C, 17020B - 17021B		
Asthma-related test and examinations	12031C, 30021C-30022C, 30005B-30006B, 30009B-30010B, 30023B-30024B		
**Chest rehabilitation and antibiotic**			
Physiotherapy	42001～42019	Pre & post chest operation Bronchiectasis Asthma	
Antibiotic	J01A<br>J01B<br>J01C<br>J01D<br>J01E	Tetracyclines<br>Amphenicols<br>Penicillins<br>Other beta-lacta<br>Cephalosporins	

	J01F<br>J01G<br>J01M<br>J01X	Macrolides<br>Aminoglycosides<br>Quinolones<br>OTHER	
Psychological therapy	45010C<br><br>45087C 45088C 45089C<br>45013C<br>45090C 45091C<br>45037C<br><br>45016C<br><br>45019C<br><br>Related procedure<br>45061C 45094C<br>45037A、5038B、45039C<br>45034A, 45035B, 45036C<br>45079C、45080B 45081C<br>45046C、45098C 45099C<br>45055C、45056B、4505*4C*<br>*45040C*、*45041B 45042C*	Supportive individual Psychotherapy<br>Re-educative individual psychotherapy<br>Intensive individual psychotherapy<br>Psychiatric inpatient special care<br>Supportive group psychotherapy<br>Intensive group psychotherapy	

Supplemental Table S2. Incidence of Arrhythmia by age, sex, comorbidity, and medication and Cox model measured hazards ratio according to medication status

	GLP-1RA							
	No			Yes				
Variables	Event	PY	Rate[#]	Event	PY	Rate[#]	Crude SHR (95% CI)	Adjusted SHR[a] (95% CI)
Age, years								
≤ 49	84	19308	4.35	111	19508	5.69	1.31(0.99, 1.74)	1.34(1.01, 1.79)*
50-64	113	18913	5.97	158	18143	8.71	1.47(1.16, 1.88)**	1.46(1.14, 1.86)**
≧65	74	7231	10.2	85	6958	12.2	1.22(0.89, 1.67)	1.25(0.91, 1.72)
p for interaction								0.63
Gender								
Women	172	24832	6.93	207	24593	8.42	1.23(1.00, 1.50)*	1.25(1.02, 1.53)*
Men	99	20620	4.80	147	20016	7.34	1.54(1.19, 1.99)***	1.53(1.18, 1.97)**
p for interaction								0.55
Comorbidity[‡]								
No	5	1337	3.74	9	1118	8.05	2.31(0.79, 6.75)	2.31(0.75, 7.06)
Yes	226	44115	6.03	345	43490	7.93	1.33(1.13, 1.56)***	1.34(1.14, 1.58)***

p for interaction								0.00
Medications								
AGI								
No	106	19449	5.45	131	18838	6.95	0.89(0.68, 1.17)	1.31(1.01, 1.69)*
Yes	165	26003	6.35	223	25770	8.65	0.62(0.50, 0.77)***	1.38(1.13, 1.69)**
p for interaction								0.98
Metformin								
No	3	181	16.6	6	331	18.2	1.20(0.32, 4.53)	0.57(0.10, 3.27)
Yes	368	45271	5.92	348	44278	7.86	1.34(1.14, 1.57)***	1.36(1.16, 1.60)***
p for interaction								0.82
Insulin								
No	35	7546	4.64	48	7352	6.53	1.42(0.92, 2.19)	1.57(1.01, 2.44)*
Yes	236	37906	6.23	306	37256	8.21	1.33(1.12, 1.58)***	1.34(1.13, 1.59)***
p for interaction								0.93
DPP-4 inhibitor								
No	39	5063	7.70	41	5178	7.92	1.03(0.66, 1.60)	1.10(0.71, 1.71)
Yes	232	40389	5.74	313	39431	7.94	1.40(1.18, 1.65)***	1.38(1.17, 1.64)***
p for interaction								0.52
Meglitinides								
No	183	31268	5.85	232	30231	7.67	1.32(1.09, 1.60)**	1.33(1.10, 1.62)**
Yes	88	14184	6.20	122	14378	8.49	1.39(1.06, 1.83)*	1.39(1.06, 1.83)*
p for interaction								0.97
TZD								
No	113	16495	6.85	128	16255	7.87	1.16(0.90, 1.49)	1.18(0.91, 1.53)
Yes	158	28957	5.46	226	28353	7.97	1.48(1.21, 1.81)***	1.48(1.20, 1.81)***
p for interaction								0.93
Sulphonylurea								
No	16	2839	5.64	24	3281	7.31	1.30(0.69, 2.45)	1.38(0.74, 2.58)
Yes	255	42613	5.98	330	41327	7.99	1.35(1.15, 1.59)***	1.35(1.15, 1.60)***
p for interaction								0.50
Statin								
No	33	5686	5.80	55	5851	9.40	1.65(1.07, 2.54)*	1.77(1.15, 2.72)**
Yes	238	39766	5.99	299	38758	7.71	1.30(1.10, 1.54)**	1.32(1.11, 1.57)**
p for interaction								0.50
Antidepressants								
No	158	30086	5.25	212	29088	7.29	1.40(1.14, 1.72)**	1.42(1.15, 1.74)***
Yes	113	15366	7.35	142	15521	9.15	1.26(0.98, 1.61)	1.26(0.98, 1.62)
p for interaction								0.67
Antihypertensive								
No	13	5518	2.36	23	5680	4.05	1.72(0.87, 3.38)	1.57(0.81, 3.04)
Yes	258	39934	6.46	331	38928	8.50	1.33(1.13, 1.57)***	1.34(1.14, 1.58)***
p for interaction								0.89
Antithrombotic								

No	97	23960	4.05	123	23889	5.15	1.28(0.98, 1.67)	1.28(0.98, 1.68)
Yes	174	21492	8.10	231	20792	11.2	1.40(1.15, 1.70)***	1.40(1.15, 1.70)**
p for interaction								0.95
Psychological therapy								
No	154	33410	5.11	232	22129	7.56	1.43(1.23,1.77) **	1.44(1.54,1.99) **
Yes	110	15231	7.23	140	14231	10.3	1.32(0.98,1.90)	1.39(0.99,1.90)
p for interaction								0.01

HR=Hazard ratio; CI=Confidence Interval; PY= Persona year
Rate[#], incidence rate, per 1,000 person-years; Crude HR, relative hazard ratio; [a]
Adjusting for age, gender, comorbidities of hypertension, hyperlipidemia, chronic renal disease, gout, tobacco dependence-related, venous thrombosis. depression or substance related and medications of alpha glucosidase inhibitor (AGI), metformin, insulin, dipepidyl peptidase 4 (DPP-4) inhibitor, meglitinides, thiazolidinedione (TZD), sulphonylurea, statin, antidrepressants, antihypertensive, antithrombotic and psychological therapy
Comorbidity[‡]: Patients with any one of the comorbidities listing in Table 1 as the comorbidity group
*p<0.05, **p<0.01, ***p<0.001

Supplemental Table S3. Incidence and adjusted hazard ratio of Arrhythmia stratified by cumulative use day per year and cumulative dose per year of GLP-1RA therapy

Medication exposed	N	Event	Person-year	Rate	Crude SHR(95% CI)	Adjusted SHR(95% CI)[a]
GLP-1RA						
No	15801	271	45452	5.96	1.00	1.00
Yes[#]						
< 85 days	4097	125	10946	11.4	1.91(1.54, 2.36)***	1.79(1.45, 2.22)***
86-200 days	3719	64	8910	7.18	1.23(1.01, 1.61) *	1.24(1.02, 1.63) *
201-350 days	3961	50	9688	5.16	1.09(1.02, 1.20) *	1.15(1.03, 1.24) *
>=351 days	4024	90	15064	5.12	1.02(0.90, 1.60)	1.01(0.98, 1.68)

[#]The cumulative use day per year are partitioned in to 4 segments by quartile
[a] Adjusting for age, gender, comorbidities of hypertension, hyperlipidemia, chronic renal disease, gout, tobacco dependence-related, venous thrombosis. depression or substance related and medications of alpha glucosidase inhibitor (AGI), metformin, insulin, dipepidyl peptidase 4 (DPP-4) inhibitor, meglitinides, thiazolidinedione (TZD), sulphonylurea, statin, antidrepressants, antihypertensive, antithrombotic and psychological therapy
Abbreviations: aSHR, adjusted subhazard ratio;
*p<0.05, **p<0.01, ***p<0.001
